# Acquired Factor XIII Deficiency Is Common during ECMO Therapy and Associated with Major Bleeding Events and Transfusion Requirements

**DOI:** 10.3390/jcm12124115

**Published:** 2023-06-18

**Authors:** Matthias Noitz, Roxane Brooks, Johannes Szasz, Dennis Jenner, Carl Böck, Niklas Krenner, Martin W. Dünser, Jens Meier

**Affiliations:** 1 Department of Anesthesiology and Intensive Care Medicine, Kepler University Hospital GmbH, Johannes Kepler University Linz, Krankenhausstraße 9, 4020 Linz and Altenberger Strasse 69, 4040 Linz, Austria; 2 Institute of Signal Processing, Johannes Kepler University Linz, Altenberger Strasse 69, 4040 Linz, Austria; 3 Department of Cardiac, Vascular and Thoracic Surgery, Kepler University Hospital GmbH, Johannes Kepler University Linz, Krankenhausstraße 9, 4020 Linz and Altenberger Strasse 69, 4020 Linz, Austria

**Keywords:** ECMO, factor XIII, deficiency, bleeding, red blood cells, transfusion

## Abstract

Background: Bleeding events are frequent complications during extracorporeal membrane oxygenation therapy (ECMO). Objective: To determine the rate of acquired factor XIII deficiency and its association with major bleeding events and transfusion requirements in adults undergoing ECMO therapy. Materials and Methods: A retrospective single centre cohort study. Adult patients receiving veno-venous or veno-arterial ECMO therapy during a 2-year period were analysed and screened for factor XIII activity measurements. Factor XIII deficiency was defined based on the lowest factor XIII activity measured during ECMO therapy. Results: Among 84 subjects included into the analysis, factor XIII deficiency occurred in 69% during ECMO therapy. There were more major bleeding events (OR, 3.37; 95% CI, 1.16–10.56; *p* = 0.02) and higher transfusion requirements (red blood cells, 20 vs. 12, *p* < 0.001; platelets, 4 vs. 2, *p* = 0.006) in patients with factor XIII deficiency compared to patients with normal factor XIII activity. In a multivariate regression model, factor XIII deficiency was independently associated with bleeding severity (*p* = 0.03). Conclusions: In this retrospective single centre study, acquired factor XIII deficiency was observed in 69% of adult ECMO patients with a high bleeding risk. Factor XIII deficiency was associated with higher rates of major bleeding events and transfusion requirements.

## 1. Introduction

Haemostatic complications (thrombotic and haemorrhagic) are frequent during ECMO therapy and adversely impact on morbidity and mortality [1,2,3,4,5]. Up to 30–60% of ECMO patients were found to suffer from at least one significant bleeding event [2,6,7,8]. This increased bleeding risk results from the need for systemic anticoagulation to prevent clot formation in the ECMO circuit, as well as coagulation disorders induced by shear stress and contact of blood with negatively charged surfaces [9,10]. Coagulation disorders during ECMO therapy involve both the cellular (e.g., platelet activation and consumption, platelet dysfunction) and plasmatic (e.g., acquired von Willebrand Disease, consumption of coagulation factors) coagulation system [11,12,13,14,15].

Due to the high morbidity caused by haemostatic complications, several strategies have been tested to reduce the risk of bleeding in ECMO patients. Alternative drugs for systemic anticoagulation such as bivalirudin showed a reduced mortality in adult patients and a significant reduction in transfusion requirements during the first 24 h of ECMO support in paediatric patients compared to those receiving unfractionated heparin (UFH) [16]. Nafamostat mesilate (NM), a synthetic serine protease inhibitor, might be a possible future alternative anticoagulation option for patients with high bleeding risk or side-effects from other anticoagulants. However, prospective studies on the use of NM are lacking and more data is needed to evaluate its role in ECMO anticoagulation [17]. Furthermore, studies on complete omission of systemic anticoagulation in patients undergoing ECMO have been evaluated in a systematic review showing comparable rates of circuit and patient thrombosis in subjects both with and without systemic anticoagulation, a finding particularly clinically relevant for patients with a high borderline bleeding risk or active bleeding complications (e.g., intracerebral haemorrhage, etc.) [18]. Routine viscoelastic point-of-care (POC) tests such as rotational thromboelastometry, thromboelastography, and platelet function testing, though able to support the detection of surgical bleeding, have failed to reduce both bleeding and thrombosis events in ECMO patients [19].

Coagulation factor XIII is activated by thrombin and calcium and mediates the cross-linking of fibrin, thereby stabilizing the clot and protecting it from premature enzymatic degradation [20]. Factor XIII deficiency has been associated with delayed haemorrhagic complications and the need for surgical re-exploration following cardiac surgery as well as bleeding complications in neurosurgical patients and high morbidity in both medical and surgical populations [21,22,23,24]. Acquired factor XIII deficiency has been reported during ECMO therapy in small populations of adults and children [14,25,26,27]. However, no study has so far evaluated the association between the rate and severity of factor XIII deficiency, major bleeding and transfusion needs during ECMO therapy.

In this explorative study, we sought to determine the rate of factor XIII deficiency and its association with major bleeding events and transfusion requirements in adults undergoing ECMO therapy.

## 2. Materials and Methods

This analysis was designed as a single centre, retrospective cohort study and conducted at the Department of Anaesthesiology and Intensive Care Medicine at the Kepler University Hospital, a tertiary hospital and ECMO referral centre, in Linz/Austria. The study protocol was reviewed and approved by the institutional Ethics committee (Ethikkommission der Medizinischen Fakultät der Johannes Kepler Universität; Reference Number: EK1210/2022). Due to the retrospective nature of the protocol, written informed consent was waived.

### 2.1. Coagulation Management during ECMO Therapy

At the study centre, ECMO therapy was performed using the XENIOS*^®^* system (Xenios AG*^®^*; Fresenius Medical Care Company, Heilbronn, Germany), a multi-functional, full extracorporeal life support system console able to assist in both heart and lung support, and albumin-heparin coated circuits (X.ellence*^®^*; Novalung IPS Kits, Fresenius Medical Care Company, Heilbronn, Germany). For vascular access, albumin-heparin coated cannulas (Getinge AB*^®^*; Getinge, Göteborg, Sweden) were chosen. In case of veno-venous ECMO (vv-ECMO), a femoro-jugular cannulation technique was applied by the critical care team; in case of veno-arterial ECMO (va-ECMO), a standardized femoro-axillary cannulation with open surgical cut-down technique was performed by the cardio-thoracic surgery team. Unfractionated heparin was used as the primary mode of anticoagulation and titrated to an activated partial thromboplastin time (aPTT) between 50 and 60 s. In case this target could not be achieved despite a maximum heparin dose of 35,000 IU/day, argatroban was used instead. According to an institutional protocol, epoprostenol (0.005–0.01 mg/h) was continuously infused directly into the oxygenator. In selected cases (e.g., platelet counts <50 G/L, suspected platelet dysfunction, excessive bleeding risk, active bleeding after instillation of ECMO therapy) epoprostenol infusion was withheld. Based on the institutional ECMO-anticoagulation protocol, extensive coagulation studies including aPTT, prothrombin time, fibrinogen, platelets and D-dimer were performed at 8 h intervals in all ECMO patients. In selected cases (e.g., bleeding events or difficulties in adjusting the rate of unfractionated heparin to the desired therapeutic range), the multi-professional critical care team could deviate from this protocol by modulation of both standard anticoagulation drugs and the time intervals of coagulation studies.

ECMO circuits were changed in case of macroscopic clotting in the circuit or oxygenator, or if there was laboratory evidence of systemic fibrinolysis and/or haemolysis presumed to be induced by the ECMO circuit or oxygenator on the basis of deteriorating oxygenator function, increases in trans-oxygenator pressure, rising D-dimer levels, plasma free haemoglobin and/or lactate dehydrogenase serum concentrations.

Factor XIII activity was not routinely measured in ECMO patients but determined at the discretion of the attending multidisciplinary critical care team in patients with a high risk for bleeding complications or with actual signs of bleeding. For measurements of factor XIII activity, a photometric assay (Berichrom^®^ FXIII; Siemens Healthcare Diagnostics, Marburg, Germany) was used.

Furthermore, in case of bleeding, viscoelastic tests (ROTEM sigma; Werfen GmbH, Vienna, Austria) were performed at the discretion of the attending multidisciplinary critical care team. Packed red blood cells (pRBCs) were transfused to target haematocrit values of 30% in patients during ECMO support.

### 2.2. Inclusion and Exclusion Criteria

All patients receiving either veno-venous or veno-arterial ECMO therapy at the study centre during the time from January 2020 until December 2021 were eligible for study enrolment. Patients <18 years, in whom no factor XIII activity was measured during ECMO therapy, and subjects with a pre-existing or inherited factor XIII deficiency were excluded from the analysis.

### 2.3. Data Collection

The following data were extracted from the electronic records of all study patients: demographic data, indication for ECMO therapy, ECMO configuration, cannula sizes, anticoagulation strategy, duration of ECMO therapy, length of intensive care unit stay and intensive care unit, as well as hospital mortality. During ECMO therapy, the lowest factor XIII activity, the minimum and maximum prothrombin time, partial thromboplastin time, fibrinogen levels, D-dimer levels, platelet counts and minimum haematocrit concentration, as well as major bleeding complications, requirements for packed red blood cells, fresh frozen plasma and platelet transfusion and fibrinogen, prothrombin complex and coagulation factor XIII concentrates were recorded.

Data collection was in accordance with the “Good Scientific Practice”- and “Good Clinical Practice”- Guidelines and accomplished with an electronic database system (Microsoft Excel^®^; Microsoft Deutschland GmbH, Unterschleißheim, Germany) [28,29].

### 2.4. Study Endpoints

The primary endpoint was to determine the rate of factor XIII deficiency in study patients. The secondary endpoint was to evaluate the association between factor XIII deficiency and major bleeding complications in the study population.

### 2.5. Definitions

Factor XIII deficiency was defined based on the lowest factor XIII activity measured during ECMO therapy. In accordance with the Extracorporeal Life Support Organization, major bleeding events were defined as follows [30]: (1) a decrease in haemoglobin levels ≥ 2 g/dL over a 24 h period; (2) blood loss greater than 20 mL/kg over a period of 24 h; (3) requirement of 2 or more packed red blood cell transfusions over that same time period; (4) retroperitoneal, pulmonary or central nervous system bleeding; or (5) any bleeding requiring surgical intervention.

### 2.6. Statistical Analysis

Following the locking of the database and plausibility control of entered values, all statistical analyses were performed, and figures drafted using the R^®^ statistical software version 4.2.0 (R^®^ Core Development Team, Vienna, Austria). No imputation method was used to compensate for missing values. Testing for normal distribution of continuous variables was conducted using the Shapiro–Wilk test. Descriptive statistical methods were applied to report demographic, clinical, laboratory and outcome data. Categorical and continuous variables were compared between patients with and without factor XIII deficiency with the use of the Fisher’s exact or Wilcoxon rank-sum test, as appropriate. A correlation analysis was applied to evaluate the relationship between factor XIII activity and the number of packed red blood cells transfused. To determine the association between factor XIII deficiency and the occurrence of one or more major bleeding events, a multivariate logistic regression model was calculated. Two-sided *p*-values < 0.05 were considered to indicate statistical significance. Data are given as absolute numbers with percentages or median values with interquartile ranges.

## 3. Results

**Figure 1:**
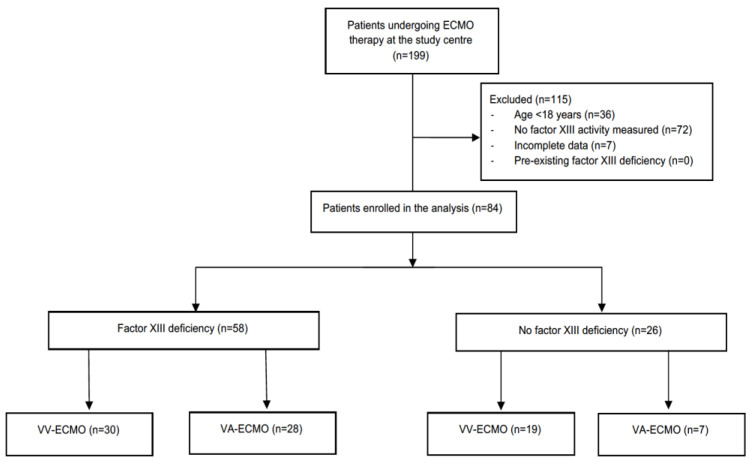
Flow diagram of database screening and study enrolment. ECMO, extracorporeal membrane oxygenation; VA, veno-arterial; VV, veno-venous.

During the observation period, 199 patients underwent ECMO therapy at the study centre and were screened for eligibility. Of these, 84 patients were included into the analysis (Figure 1).

**Figure 2:**
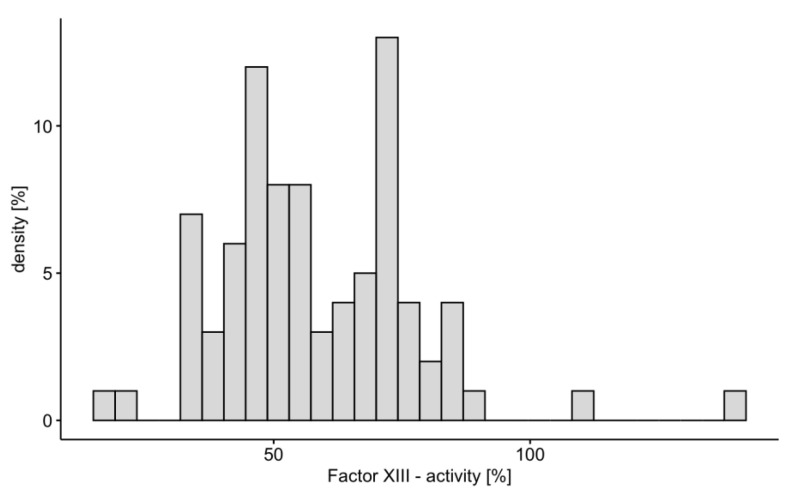
Distribution of factor XIII activity in study patients.

Fifty-eight study patients (69%) met the criteria of factor XIII deficiency (Figure 2). There were no differences in demographic data, ECMO indication and configuration, cannula size, anticoagulation strategy, duration of ECMO therapy, length of intensive care unit stay, as well as intensive care unit and in-hospital mortality between patients with and without factor XIII deficiency (Table 1). Except for minimum fibrinogen levels, coagulation studies during ECMO therapy did not differ between the two groups (Table 2). Factor XIII [median dose, 2500 IU (1250–3750)] was replaced in 38 of 58 (65.5%) of patients with an acquired factor XIII deficiency.

**Figure 3:**
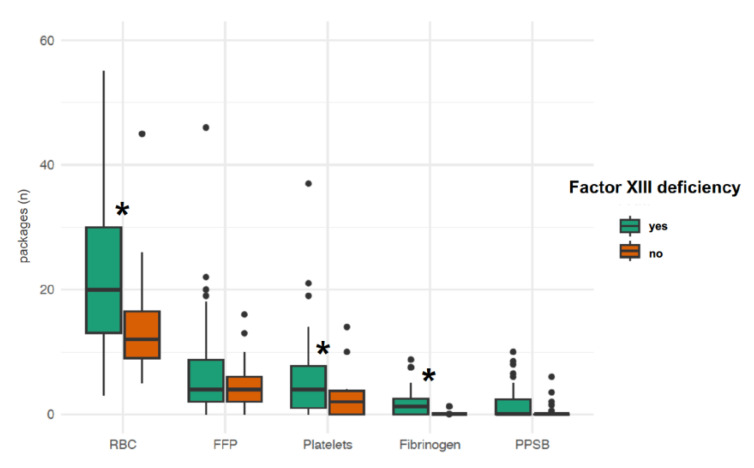
Transfusion and coagulation factor requirements in study patients with and without factor XIII deficiency. FFP, fresh frozen plasma; PPSB, prothrombin complex concentrate; RBC, red blood cell transfusion. *, significant difference between study groups.

**Figure 4:**
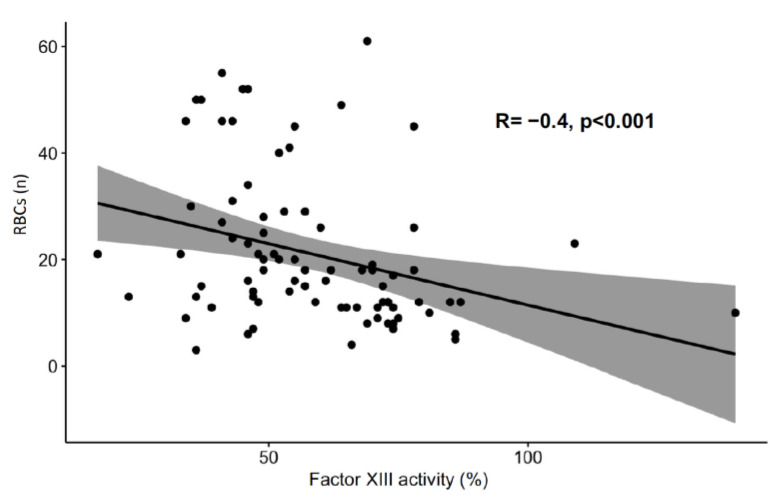
Relationship between factor XIII activity and the number of red blood cells transfused. ECMO, extracorporeal membrane oxygenation; RBC, red blood cells.

Major bleeding events occurred in 43 study patients (51.2%) during ECMO therapy (Table 3). The rate of any major bleeding event [35/58 (60.3%) vs. 8/26 (30.8%), OR, 3.37; 95% CI, 1.16–10.56; *p* = 0.02] and the median number of major bleeding events per patient [1 (0–1) vs. 0 (0–1), *p* = 0.03] were higher in subjects with factor XIII deficiency than in those without. The minimum haematocrit concentration was lower in patients with factor XIII deficiency than in those with factor XIII activity >70% [24.5% (22.6–25.6) vs. 25.9% (24.5–26.8), *p* = 0.004]. Patients with factor XIII deficiency required more packed red blood cell (*p* < 0.001) and platelet (*p* = 0.006) transfusions as well as higher fibrinogen concentrate doses (*p* = 0.01) than patients without factor XIII deficiency (Figure 3). Factor XIII activity was inversely correlated with the number of packed red blood cells transfused during ECMO therapy (Figure 4).

In a multivariate logistic regression model including age, sex, body mass index, ECMO configuration, arterial and venous cannula sizes, minimum prothrombin time, fibrinogen levels and platelet counts, maximum activated partial thromboplastin time, and the number of ECMO days, only factor XIII deficiency was significantly associated with the occurrence of one or more major bleeding events (*p* = 0.03).

## 4. Discussion

In this retrospective single centre cohort study, we detected a factor XIII deficiency in 69% of study patients during ECMO therapy. Major bleeding events occurred in 51% of subjects and were associated with a factor XIII activity <70%. In addition, factor XIII deficiency was associated with higher transfusion requirements and found to be an independent risk factor for the occurrence of major bleeding events in this study population.

The rate of factor XIII deficiency in this adult ECMO population was high. Certain aspects need to be considered when interpreting this result. Factor XIII activity was not routinely measured in all patients undergoing ECMO therapy, but only at the discretion of the attending critical care team in subjects with an increased bleeding risk or actual signs of bleeding. Due to this selection bias, the true rate of factor XIII deficiency was likely over-estimated in our cohort. However, even if taking all adult ECMO patients who were treated during the 24-month period, irrespective of whether factor XIII activities were measured or not, into account, the incidence of factor XIII deficiency during ECMO therapy would still be 37.2% (*n* = 58/156). Another potential confounder influencing the incidence of factor XIII deficiency in this study population was the fact that half of the study patients underwent ECMO therapy because of respiratory failure due to COVID-19, a condition known to be associated with acquired factor XIII deficiency [31]. Despite of these considerations, the incidence of factor XIII deficiency in our study population was comparable to previous smaller publications evaluating factor XIII activities in adults undergoing veno-venous ECMO therapy or paediatric ECMO cases [14,26,27].

Since we excluded patients with pre-existent or inherited factor XIII deficiency, we assume that all cases of factor XIII deficiency in this cohort were acquired during the disease resulting in the need for ECMO support or during ECMO therapy itself. Although no pathophysiologic explanations on the causes of factor XIII deficiency can be drawn from our study results, one may hypothesize that consumption and loss of factor XIII played a relevant role. The severity of acquired factor XIII deficiency has probably been influenced by the administration of factor XIII concentrates to 65.5% of study patients with factor XIII deficiency.

Major bleeding events occurred in 51% of study patients, a rate comparable to the current literature [2,6,7,8]. Factor XIII deficiency during ECMO therapy was associated with the occurrence and severity of major bleeding events, as indicated by higher transfusion requirements of red blood cells, platelets and fibrinogen concentrates. Although these findings do not necessarily prove a causative relationship, a multivariable regression model revealed that factor XIII deficiency was an independent risk factor for the occurrence of one or more major bleeding events in our cohort. This finding is novel and in line with reports including cardio-thoracic and neurosurgical patients, in whom factor XIII deficiency was associated with postoperative haemorrhage and higher needs for surgical re-exploration [21,22,23,32].

This study represents both the largest cohort of adult ECMO patients having been investigated for acquired factor XIII deficiency and the first study to evaluate the association between factor XIII deficiency, major bleeding events and transfusion requirements in an adult population. Due to the high morbidity caused by bleeding events and transfusion of allogeneic blood products in critically ill patients, emphasis should be placed on evaluating laboratory values or parameters that can assist in anticipation or prevention of potential bleeding complications and therefore might augment patient safety. The significant association between factor XIII deficiency and the occurrence of one or more major bleeding events, as well as higher requirements of packed red blood cells and platelet transfusions, is a novel finding in the specified group of adult patients undergoing VV-and VA-ECMO. Our results, though only hypothesis-generating in view of the retrospective nature of the study, imply that acquired factor XIII deficiency might play a key role in the complex cascade of coagulopathic changes and derangements during ECMO therapy, leading to higher bleeding associated morbidity.

However, further limitations need to be discussed when interpreting the results of our study. First and most importantly, this is a retrospective analysis, implying methodological biases such as missing values and the fact that factor XIII activity was not routinely determined during ECMO therapy. Although our study included 84 patients and thus represents the largest patient population examining factor XIII activity, bleeding events and transfusion requirements during ECMO therapy, the sample size was small and might have additionally influenced the reported rate of factor XIII deficiency in our population. Finally, as this was a single centre study, centre-specific factors such as the anticoagulation strategy applied, the type of ECMO circuits used or other treatment effects may have influenced the study endpoints reported.

## 5. Conclusions

In this retrospective single centre study, acquired factor XIII deficiency was observed in 69% of adult ECMO patients with a high bleeding risk. Factor XIII deficiency was associated with higher rates of bleeding events and transfusion requirements.

## Figures and Tables

**Table 1:** Characteristics of the study population.

		Overall	Factor XIII Deficiency	No Factor XIII Deficiency	*p* Value
***n***	(%)	84 (100)	58 (69)	26 (31)	
**Male Sex**	*n* (%)	64 (76.2)	45 (77.6)	19 (73.1)	0.78
**Age**	years	58 (49–66.3)	57 (49–65.8)	59.5 (49–66.8)	0.62
**BMI**	kg/m^2^	29.4 (26.1–34.5)	28.8 (25.6–33.9)	31.8 (27.8–35.9)	0.15
**Indications for ECMO therapy**					
*COVID-19 ARDS*	*n* (%)	43 (51.2)	28 (48.3)	15 (57.7)	0.48
*Circulatory failure*	*n* (%)	22 (26.2)	17 (29.3)	5 (19.2)	0.43
*ARDS due to bacterial pneumonia*	*n* (%)	5 (6)	2 (3.4)	3 (11.5)	0.17
*Acute myocardial ischemia*	*n* (%)	5 (6)	4 (6.9)	1 (3.8)	1
*Viral pneumonia* *(non -SARS-CoV-2)*	*n* (%)	4 (4.8)	3 (5.2)	1 (3.8)	1
*Vasculitis*	*n* (%)	1 (1.2)	0	1 (3.8)	0.31
*Decompensated valvular heart failure*	*n* (%)	1 (1.2)	1 (1.7)	0	1
*Pulmonary embolism*	*n* (%)	1 (1.2)	1 (1.7)	0	1
*COPD Exacerbation*	*n* (%)	1 (1.2)	1 (1.7)	0	1
*Pulmonary fibrosis*	*n* (%)	1 (1.2)	1 (1.7)	0	1
**Primary ECMO configuration**					
*VV-ECMO*	*n* (%)	49 (58.3)	30 (51.7)	19 (73.1)	0.09
*VA-ECMO*	*n* (%)	35 (41.7)	28 (48.3)	7 (26.9)	0.09
**ECMO configuration change**	*n* (%)	8 (9.5)	6 (10.3)	2 (7.7)	1
**Size of arterial cannula**	Fr	19 (19–19)	19 (19–19)	19 (19–20.5)	1
**Size of venous cannula**	Fr	25 (25–25)	25 (25–25)	25 (25–25)	0.06
**Anticoagulation**					
*UFH*	*n* (%)	80 (95.2)	57 (98.3)	23 (88.5)	0.09
*Argatroban*	*n* (%)	4 (4.8)	1 (1.7)	3 (11.5)	0.09
*Epoprostenol*	*n* (%)	79 (94)	56 (96.6)	23 (88.5)	0.17
**Length of ECMO therapy**	days	17 (8–27)	17 (8–28)	17 (7–24)	0.86
**ICU length of stay**	days	24 (15–31)	24 (14–30)	21 (16–34)	0.97
**ICU mortality**	*n* (%)	45 (53.6)	33 (56.9)	12 (46.2)	0.48
**In-hospital mortality**	*n* (%)	46 (54.8)	33 (56.9)	13 (50)	0.64

ARDS, acute respiratory distress syndrome; BMI, body mass index; COPD, chronic obstructive pulmonary disease; COVID-19, coronavirus disease 2019; ECMO, extracorporeal membrane oxygenation; Fr, French; ICU, intensive care unit; UFH, unfractionated heparin; SARS-CoV-2, severe acute respiratory syndrome coronavirus type 2; VA, veno-arterial; VV, veno-venous.

**Table 2:** Results of Coagulation studies in study patients.

		Overall	Factor XIII Deficiency	No Factor XIII Deficiency	*p-*Value
***n***		84	58	26	
**Minimum PT**	%	54 (38–64)	51 (37–64)	55 (46.3–63.5)	0.22
**Maximum PT**	%	56.5 (41.5–80.3)	55.5 (37.3–77.3)	59 (46.3–82)	0.45
**Minimum aPTT**	sec	23.4 (22–25.9)	23.5 (22–25.8)	23.2 (22–26)	0.61
**Maximum aPTT**	sec	60.6 (50–84.7)	63.5 (50.5–93.3)	55.9 (49.5–66.6)	0.13
**Minimum** **fibrinogen levels**	mg/dL	227 (161–350)	209 (153–291)	309 (186–425)	0.01 *
**Maximum** **fibrinogen levels**	mg/dL	900 (711–900)	887 (707–900)	900 (715–900)	0.34
**Minimum** **platelets**	G/L	58 (40–81)	57 (39–71)	68 (49–101)	0.09
**Maximum** **platelets**	G/L	238 (170–313)	223 (165–293)	273 (193–360)	0.12
**Minimum** **D-dimer**	mg/L	1.7 (1.1–3.1)	1.7 (1.1–3.5)	1.9 (1.2–2.6)	0.9
**Maximum** **D-dimer**	mg/L	35.2 (11.2–35.2)	31.8 (10.0–35.2)	35.2 (13.9–35.2)	0.14

aPTT, activated partial thromboplastin time; PT, prothrombin time; sec, seconds; *, significant difference between study groups.

**Table 3:** Bleeding sources in study patients experiencing a major bleeding event (*n* = 43).

		Factor XIII Deficiency	No Factor XIII Deficiency
***n***		35	8
**Haemopericardium**	*n* (%)	11 (25.6)	0
**Haemothorax**	*n* (%)	10 (23.3)	2 (18.2)
**Pulmonary**	*n* (%)	6 (14)	3 (27.3)
**Ear/Nose/Throat**	*n* (%)	5 (11.6)	1 (9.1)
**Cerebral**	*n* (%)	4 (9.3)	1 (9.1)
**Gastrointestinal**	*n* (%)	3 (7)	2 (18.2)
**Vascular**	*n* (%)	1 (2.3)	1 (9.1)
**Abdominal/Pelvis**	*n* (%)	2 (4.7)	0
**Others**	*n* (%)	1 (2.3)	1 (9.1)
**Total number of** **Major bleeding events**	*n* (%)	43 (79.6%)	11(20.4%)

## Data Availability

The data presented in this study are available upon a reasonable request from the corresponding author. The data are not publicly available due to GDPR and local regulatory/data privacy provisions.

## References

[1] Aubron C., Cheng A.C., Pilcher D., Leong T., Magrin G., Cooper D.J., Scheinkestel C., Pellegrino V. (2013). Factors associated with outcomes of patients on extracorporeal membrane oxygenation support: A 5-year cohort study. Crit. Care.

[2] Mazzeffi M., Greenwood J., Tanaka K., Menaker J., Rector R., Herr D., Kon Z., Lee J., Griffith B., Rajagopal K. (2016). Bleeding, Transfusion, and Mortality on Extracorporeal Life Support: ECLS Working Group on Thrombosis and Hemostasis. Ann. Thorac. Surg..

[3] Vaquer S., de Haro C., Peruga P., Oliva J.C., Artigas A. (2017). Systematic review and meta-analysis of complications and mortality of veno-venous extracorporeal membrane oxygenation for refractory acute respiratory distress syndrome. Ann. Intensive Care.

[4] Zangrillo A., Landoni G., Biondi-Zoccai G., Greco M., Greco T., Frati G., Patroniti N., Antonelli M., Pesenti A., Pappalardo F. (2013). A meta-analysis of complications and mortality of extracorporeal membrane oxygenation. Crit. Care Resusc..

[5] Thomas J., Kostousov V., Teruya J. (2018). Bleeding and Thrombotic Complications in the Use of Extracorporeal Membrane Oxygenation. Semin. Thromb. Hemost..

[6] Aubron C., DePuydt J., Belon F., Bailey M., Schmidt M., Sheldrake J., Murphy D., Scheinkestel C., Cooper D.J., Capellier G. (2016). Predictive factors of bleeding events in adults undergoing extracorporeal membrane oxygenation. Ann. Intensive Care.

[7] Willers A., Swol J., Buscher H., McQuilten Z., van Kuijk S.M.J., Ten Cate H., Rycus P.T., McKellar S., Lorusso R., Tonna J.E. (2022). Longitudinal Trends in Bleeding Complications on Extracorporeal Life Support Over the Past Two Decades—Extracorporeal Life Support Organization Registry Analysis. Crit. Care Med..

[8] Nunez J.I., Gosling A.F., O’Gara B., Kennedy K.F., Rycus P., Abrams D., Brodie D., Shaefi S., Garan A.R., Grandin E.W. (2022). Bleeding and thrombotic events in adults supported with venovenous extracorporeal membrane oxygenation: An ELSO registry analysis. Intensive Care Med..

[9] Doyle A.J., Hunt B.J. (2018). Current Understanding of How Extracorporeal Membrane Oxygenators Activate Haemostasis and Other Blood Components. Front. Med..

[10] Kalbhenn J., Zieger B. (2022). Bleeding During Veno-Venous ECMO: Prevention and Treatment. Front. Med..

[11] Tauber H., Ott H., Streif W., Weigel G., Loacker L., Fritz J., Heinz A., Velik-Salchner C. (2015). Extracorporeal membrane oxygenation induces short-term loss of high-molecular-weight von Willebrand factor multimers. Anesth. Analg..

[12] Kalbhenn J., Schlagenhauf A., Rosenfelder S., Schmutz A., Zieger B. (2018). Acquired von Willebrand syndrome and impaired platelet function during venovenous extracorporeal membrane oxygenation: Rapid onset and fast recovery. J. Heart Lung Transpl..

[13] Kalbhenn J., Schmidt R., Nakamura L., Schelling J., Rosenfelder S., Zieger B. (2015). Early diagnosis of acquired von Willebrand Syndrome (AVWS) is elementary for clinical practice in patients treated with ECMO therapy. J. Atheroscler. Thromb..

[14] Kalbhenn J., Wittau N., Schmutz A., Zieger B., Schmidt R. (2015). Identification of acquired coagulation disorders and effects of target-controlled coagulation factor substitution on the incidence and severity of spontaneous intracranial bleeding during veno-venous ECMO therapy. Perfusion.

[15] Kalbhenn J., Glonnegger H., Wilke M., Bansbach J., Zieger B. (2021). Hypercoagulopathy, acquired coagulation disorders and anticoagulation before, during and after extracorporeal membrane oxygenation in COVID-19: A case series. Perfusion.

[16] Seelhammer T.G., Bohmann J.K., Schulte P.J., Hanson A.C., Aganga D.O. (2021). Comparison of Bivalirudin Versus Heparin for Maintenance Systemic Anticoagulation During Adult and Pediatric Extracorporeal Membrane Oxygenation. Crit. Care Med..

[17] Sanfilippo F., Currò J.M., La Via L., Dezio V., Martucci G., Brancati S., Murabito P., Pappalardo F., Astuto F. (2022). Use of nafamostat mesylate for anticoagulation during extracorporeal membrane oxygenation: A systematic review. Artif. Organs..

[18] Olson S.R., Murphree C.M., Zonies D., Meyer A.D., Maccarthy O.J.T., Deloughery T.G., Shatzel J.J. (2021). Thrombosis and Bleeding in Extracorporeal Membrane Oxygenation (ECMO) Without Anticoagulation: A Systematic Review. Asaio J..

[19] Jiritano F., Fina D., Lorusso R., Ten Cate H., Kowalewski M., Matteucci M., Serra R., Mastroroberto P., Serraino G.F. (2021). Systematic review and meta-analysis of the clinical effectiveness of point-of-care testing for anticoagulation management during ECMO. J. Clin. Anesth..

[20] Muszbek L., Bereczky Z., Bagoly Z., Komáromi I., Katona É. (2011). Factor XIII: A Coagulation Factor With Multiple Plasmatic and Cellular Functions. Physiol. Rev..

[21] Blome M., Isgro F., Kiessling A.H., Skuras J., Haubelt H., Hellstern P., Saggau W. (2005). Relationship between factor XIII activity, fibrinogen, haemostasis screening tests and postoperative bleeding in cardiopulmonary bypass surgery. Thromb. Haemost..

[22] Adam E.H., Meier J., Klee B., Zacharowski K., Meybohm P., Weber C.F., Pape A. (2020). Factor XIII activity in patients requiring surgical re-exploration for bleeding after elective cardiac surgery—A prospective case control study. J. Crit. Care.

[23] Gerlach R., Raabe A., Zimmermann M., Siegemund A., Seifert V. (2000). Factor XIII deficiency and postoperative hemorrhage after neurosurgical procedures. Surg. Neurol..

[24] Duque P., Chasco-Ganuza M., Ortuzar A., Almaraz C., Terradillos E., Perez-Rus G., Pascual C. (2022). Acquired FXIII Deficiency is Associated with High Morbidity. Thromb. Haemost..

[25] Ito A., Iwashita Y., Esumi R., Sasaki K., Yukimitsu M., Kato T., Kawamoto E., Suzuki K., Imai H. (2020). Acquired factor XIII deficiency in two patients with bleeding events during veno-venous extracorporeal membrane oxygenation treatment. J. Artif. Organs.

[26] Sanchez D., Stine K., Crary S.E., Fiser R., Schmitz M., Prodham P., Nick T., Tang X. (2013). A Pilot Study To Examine The Effect Of Extracorporeal Membrane Oxygenation (ECMO) On Plasma Factor XIII Levels. Blood.

[27] Moerer O., Huber-Petersen J.F., Schaeper J., Binder C., Wand S. (2021). Factor XIII Activity Might Already Be Impaired before Veno-Venous ECMO in ARDS Patients: A Prospective, Observational Single-Center Cohort Study. J. Clin. Med..

[28] World Medical Association (2013). World Medical Association Declaration of Helsinki: Ethical principles for medical research involving human subjects. JAMA.

[29] European Medicines Agency (EMA) (2016). ICH E6 (R2) Good Clinical Practice—Scientific Guideline. https://www.ema.europa.eu/en/documents/scientific-guideline/ich-guideline-good-clinical-practice-e6r2-step-5_en.pdf.

[30] Laurance Lequier G.A., Al-Ibrahim O., Bembea M., Brodie D., Brogan T., Buckvold S., Chicoine L., Conrad S., Cooper D., Dalton H., Extracorporeal Life Support Organization (2014). ELSO Anticoagulation Guideline.

[31] von Meijenfeldt F.A., Havervall S., Adelmeijer J., Lundström A., Magnusson M., Mackman N., Thalin C., Lisman T. (2021). COVID-19 is associated with an Acquired Factor XIII Deficiency. Thromb. Haemost..

[32] Gerlach R., Tölle F., Raabe A., Zimmermann M., Siegemund A., Seifert V. (2002). Increased risk for postoperative hemorrhage after intracranial surgery in patients with decreased factor XIII activity: Implications of a prospective study. Stroke.

